# Internal defect database of mechanically deformed ferritic steel via X-ray computed tomography

**DOI:** 10.1038/s41597-025-06141-y

**Published:** 2025-11-28

**Authors:** Gunjick Lee, Gyeong Hoon Yi, Leslie Ching Ow Tiong, Seok Su Sohn, Donghun Kim

**Affiliations:** 1 Department of Materials Science and Engineering, Korea University, Seoul, 02841 Republic of Korea; 2 Center for Hydrogen Energy Materials, Korea Institute of Science and Technology (KIST), Seoul, 02792 Republic of Korea; 3 Computational Science Research Center, Korea Institute of Science and Technology (KIST), Seoul, 02792 Republic of Korea; 4 Semiconductor R&D Center, Samsung Electronics Co., Ltd., Hwaseong, 18448 Republic of Korea; 5 Department of Materials Science and Engineering, Korea Advanced Institute of Science and Technology (KAIST), Daejeon, 34141 Republic of Korea

**Keywords:** Metals and alloys, Characterization and analytical techniques

## Abstract

Steel is widely utilized as a structural material due to its favorable mechanical properties, cost-effectiveness, and reliability, and it is expected to remain critical in engineering applications. The properties and durability of steel are significantly influenced by internal defects formed during mechanical deformation. Quantitative analysis of the formation and evolution of these defects is essential for developing materials that are both sustainable and tailored to specific applications. However, acquiring statistically meaningful datasets of internal defects is complex, time-consuming, and costly, resulting in a scarcity of comprehensive studies. In this study, we present two comprehensive datasets obtained from mechanically deformed ferritic steel samples using X-ray computed tomography (X-CT): (1) tensile deformation and (2) fatigue deformation. The database includes detailed quantitative descriptions of 938 defect features from 134 tensile-tested samples and 2,305 defect features from 142 fatigue-tested samples. Each dataset comprises high-resolution X-CT images and quantified internal defect metrics, facilitating detailed statistical analysis.

## Background & Summary

Steel has been extensively employed in structural applications due to its robust mechanical properties, economic viability, and established reliability. Despite decades of extensive research to ensure structural integrity, unexpected failures still pose significant risks, potentially causing substantial human casualties and property damage. To mitigate these risks, extensive theoretical frameworks and numerical methods have been developed to elucidate deformation mechanisms within steel.

Material deformation and eventual failure typically occur through the initiation and growth of internal defects^1–5^. Throughout mechanical loading, defect sizes and densities progressively increase as materials approach failure thresholds, and their distributions significantly evolve with deformation progression^6–8^. Understanding these phenomena requires comprehensive statistical analyses of internal defect topology, ideally under controlled experimental conditions to isolate the effects of external variables such as temperature and loading conditions^9–13^.

Non-destructive testing (NDT) methods, such as ultrasonic testing (UT), have been commonly employed to assess internal defect information in materials due to their rapid and cost-effective nature^14–21^. However, the resolution limitations of UT hinder the accurate detection of micron-scale defects^14^. Conversely, X-ray computed tomography (X-CT) offers superior resolution capabilities, effectively capturing internal defects at micron scales without specimen damage^22^. Despite its advantages, the use of X-CT for statistically significant defect analysis remains limited due to its associated time and financial costs.

Here, we compiled a comprehensive database^23^ of internal defect information in low-alloy ferritic steel subjected to mechanical deformation via tensile and fatigue testing. High-resolution X-CT scanning was performed post-deformation to collect defect data at various local strain levels for tensile specimens and at different fracture progression stages for fatigue specimens. Quantitative characterization of defect topology was conducted using topological data analysis (TDA), validated in previous research. The resulting database contains 276 datasets^23^: 134 tensile-tested and 142 fatigue-tested. Fatigue datasets^23^ are categorized into high-cycle fatigue, low-cycle fatigue, and ultralow-cycle fatigue. Each dataset^23^ includes detailed specimen information (sample ID, loading conditions, local strain, fracture progression) and defect metrics (3D X-CT images and TDA-quantified data).

This database^23^ aims to substantially contribute to the understanding and modeling of tensile and fatigue behaviors in steels. The reliability of the dataset was validated by ensuring consistency of internal defect evolution with established theoretical and experimental observations and further supported by robust clustering analyses.

## Methods

**Fig. 1:**
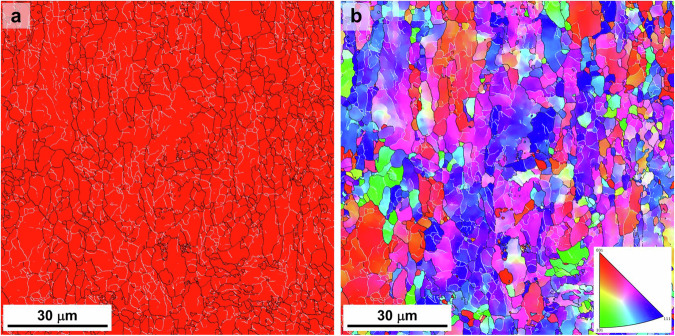
EBSD analysis of the initial microstructure of the ferritic steel. (**a**) Phase map showing that the microstructure consists entirely of the BCC phase. (**b**) IPF map of the same region. In both maps, high-angle grain boundaries (≥15°) are drawn as black lines, while low-angle boundaries (2–15°) are drawn as white lines.

**Fig. 2:**
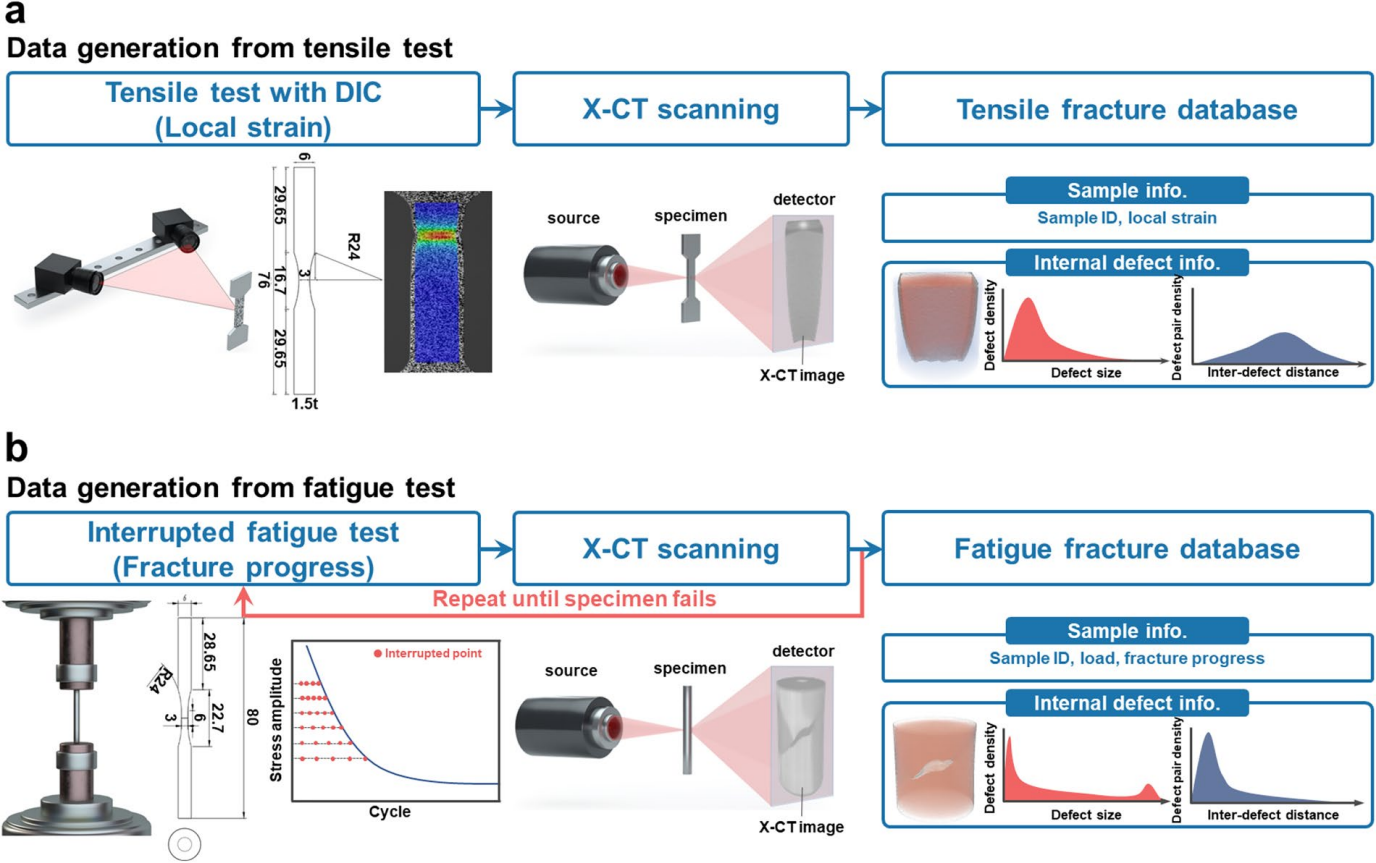
Schematic representation of the data generation workflow. (**a**) Tensile testing employed miniature plate-shaped specimens with a gauge length of 6.4 mm and thickness of 3 mm, incorporating digital image correlation (DIC) for strain measurement. (**b**) Fatigue testing utilized miniature rod-shaped specimens with a gauge length of 6 mm and radius of 3 mm. Post-testing, specimens underwent X-CT scanning within a 3 × 3 × 3 mm^3^ volume at 3 µm resolution. Each tensile dataset consists of a sample ID, local strain, 3D X-CT images, and quantified defect metrics, while fatigue datasets include sample ID, loading conditions, fracture progression, 3D X-CT images, and quantified defect metrics.

A ferritic low-alloy steel was used with the following chemical composition (wt.%): C < 0.05, Mn < 1.7, Si < 0.3, (Cr + Mo) < 0.4, and (Ti + Nb + V) < 0.15. The steel plate underwent heat treatment at 1,200–1,250 °C for 1 hour, followed by hot rolling at 800–850 °C, and subsequently cooled and held at 500–550 °C. The initial microstructure of the steel was characterized by electron backscatter diffraction (EBSD). The average grain size was determined to be 22.73 ± 4.39 µm, and a large number of subgrains were observed within the grains. The EBSD phase map (Fig. 1a) confirmed that the microstructure consists entirely of BCC phase, and no micron-scale inclusions were detected in the examined regions. The inverse pole figure (IPF) map (Fig. 1b) further revealed high-angle grain boundaries (≥15°, marked by black lines) and low-angle boundaries (2–15°, marked by white lines). Figure 2 schematically illustrates the data generation workflow. Mechanical tests were performed, and the resulting deformed specimens were scanned using a ZEISS Xradia 520 Versa X-CT system. Miniature tensile and fatigue specimens were employed due to equipment permeability constraints; specific dimensions are detailed in Fig. 2.

### Persistent homology

Persistent homology (PH), a method from topological data analysis (TDA), identifies topological features by tracking the appearance (birth) and disappearance (death) of voids or connected components during a filtration process^24,25^. The difference between death and birth, termed lifetime, reflects spatial properties and was interpreted, following previous work^26^, in terms of intervoid distances and distribution heterogeneity. In this study, PH was applied to binary-processed 3D X-CT images to extract descriptors including defect density, size distribution, and heterogeneity indices^27^. These descriptors were subsequently used in statistical analysis and clustering.

### Tensile testing

Figure 2a illustrates the tensile data collection workflow and specimen dimensions. Tensile tests were performed at room temperature with a strain rate of 10−³ s−¹, and local strain measurements were recorded using digital image correlation (DIC). After tensile testing to fracture, each specimen underwent X-CT acquisition 3 × 3 × 3 mm³ volumes near specimen fracture surface. The reconstructed 3D volume was partitioned into 4–5 contiguous sub-volumes, and each sub-volume was treated as one dataset and assigned a local strain value from DIC (spatial average over the corresponding region).

### Fatigue testing

Figure 2b details the fatigue testing workflow and specimen dimensions. Fatigue tests were conducted with an R-ratio of 0.1 and maximum stress amplitudes ranging from 630–680 MPa. Tests were periodically interrupted to perform X-CT scans under conditions identical to tensile tests but focused on the center gauge section. Following each scan, fatigue testing resumed until specimen failure.

**Fig. 3:**
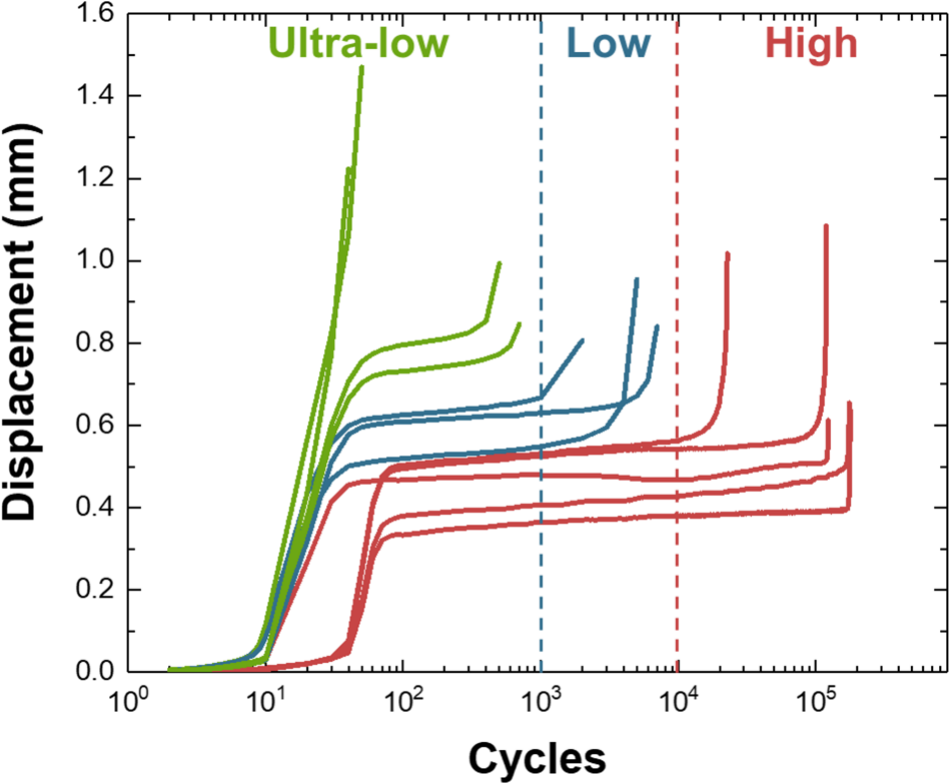
The cycle-displacement (δ) curves from fatigue testing. This plot highlights displacement stability phases corresponding to different fatigue life categories. High-cycle specimens exhibit stability between δ = 0.50–0.61 mm, while low-cycle fatigue specimens stabilize around δ = 0.71 mm. Ultralow-cycle fatigue specimens display no stable regions even beyond δ = 0.71 mm.

The initial interruption points for fatigue testing were determined via statistical analysis of displacement (δ) trends versus cycle number, as depicted in Fig. 3. Initially, displacement increased gradually before stabilizing; it subsequently rose sharply as failure approached. Interruption points were defined as follows:

- If the curve exhibited a stable displacement region at δ = 0.50 mm, this was selected as the first interruption point for high-cycle fatigue specimens (failure after >100,000 cycles).
- If no stable region was observed until δ = 0.50 mm, testing continued to δ = 0.61 mm. At this displacement, if a stable region emerged, it was designated as the first interruption point for high-cycle fatigue specimens typically failing between 10,000 and 100,000 cycles.
- If a stable area was still absent until δ = 0.61 mm, testing proceeded to δ = 0.71 mm. At this displacement, if the curve showed a stable region, it marked the first interruption point for low-cycle fatigue specimens (failure in less than 10,000 cycles). Otherwise, δ = 0.71 mm was designated as the first interruption point for ultralow-cycle fatigue specimens (failure in less than 1,000 cycles).

The rationale behind employing this multi-tiered method to determine the initial interruption point stems from the substantial variability in fracture cycles observed in specimens under different stress conditions. Due to this significant variation, the conventional approach of determining interruption timing based on S–N curves was deemed impractical. In fatigue testing, internal defect evolution becomes meaningfully detectable only after a sufficient number of load cycles. Therefore, this tailored method was developed to reliably identify a point at which a considerable level of fracture progression had occurred. Statistical analyses confirmed that the selected initial interruption points corresponded to approximately 80% of the total fracture life. Following this first interruption and the subsequent X-CT scanning, additional fatigue testing was conducted to achieve an estimated 5% further progression toward failure. This increment was calculated under the assumption that the initial interruption represented 80% of the total fracture evolution, thereby ensuring consistent and meaningful sampling of internal defect development across fatigue regimes.

## Data Records

**Fig. 4:**
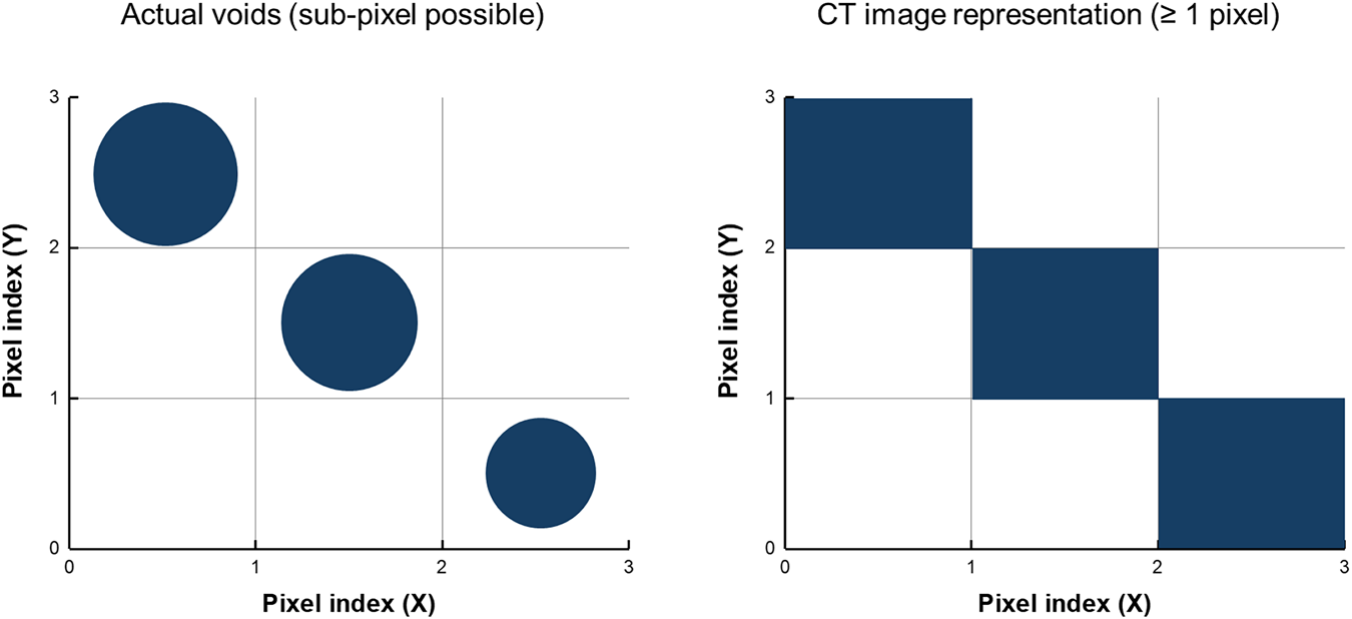
Illustration of the resolution limit in X-CT. (Left) Actual voids that can be smaller than the voxel dimension (3 µm). Such sub-pixel voids may exist within a single voxel. (Right) In CT images, these sub-pixel voids are inevitably represented as at least one pixel (3 µm), leading to an overestimation of their actual size.

The dataset is available at Dryad^23^ (10.5061/dryad.9cnp5hqpf), and contains raw X-CT volumes, metadata files, and derived descriptors of internal defects in mechanically deformed ferritic steel. Tensile datasets^23^ were stratified into seven groups based on local strain intervals of 20%, ranging from 0% to above 120%. The scans were acquired at a resolution of 3 µm, which defines the minimum detectable void size. However, voids smaller than this threshold may still appear as a single voxel in the X-CT image (Fig. 4), leading to an apparent overestimation of their actual size. To reflect this limitation, defect sizes were reported as ranges rather than single values.

Fatigue datasets^23^ were also divided into seven groups, but according to fracture progress. For fracture progress below 80%, the grouping intervals are 20%, while above 80%, finer intervals were adopted. This classification was designed to reflect the increasing sensitivity of internal defect evolution at higher levels of fatigue damage. While the raw X-CT at the same 3 µm resolution as tensile datasets^23^, the quantitative evaluations were performed on downscaled data with an effective voxel size of 12 µm due to software memory limitations. Accordingly, the minimum detectable void size in the analyzed fatigue dataset^23^ was 12 µm. In earlier stages of fatigue (fracture progress <80%), significant changes in defect characteristics were rarely observed, while at higher fracture progress levels, the structural evolution of internal defects accelerated. Therefore, broader intervals were applied at early stages, and narrower intervals at advanced stages of fatigue.

The dataset^23^ is structured into two components: a data folder and an Excel summary sheet. The data folder includes core experimental outputs for each sample, such as X-CT images and defect analysis files, providing a foundation for diverse downstream investigations. However, to supplement and facilitate accessibility, key metadata from each sample, comprising 16 analyzed parameters, has been compiled into a searchable Excel file. This sheet contains detailed descriptors of internal defects, including crack size, quantity, volume, and morphology. Each entry is linked by sample ID to the corresponding data folder, ensuring seamless navigation between summary data and raw content.

### Data folder

**Fig. 5:**
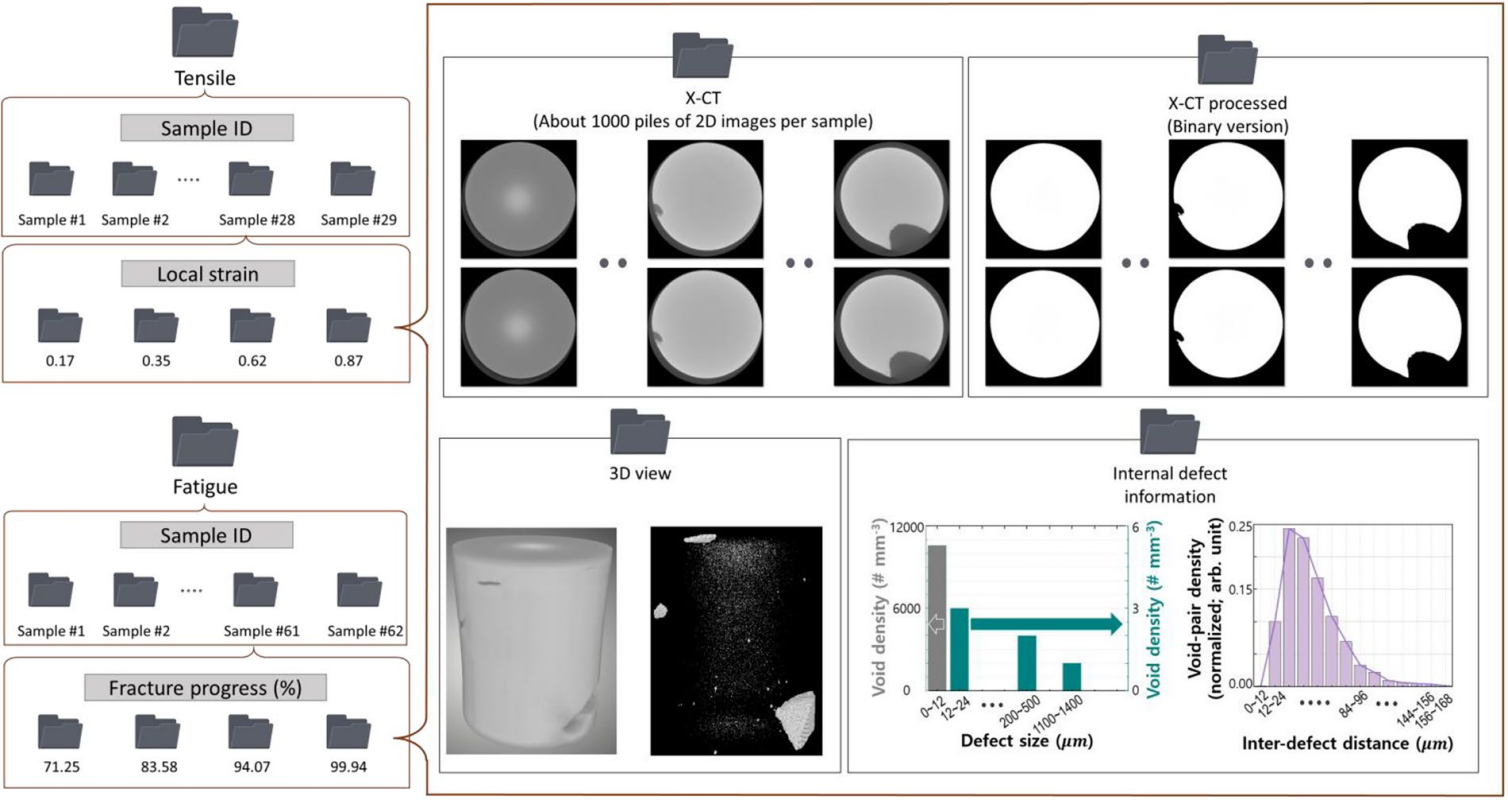
Organization of the dataset. The tensile and fatigue collections each contain approximately 150 samples, further subdivided into >250 data entries according to strain or fracture progress. Each entry includes raw and binary X-CT images, 3D views, and quantified internal defect information.

As shown in Fig. 5 the data folder is structured according to specimen type and loading mode. The top-level directions are organized as Tensile_[specimen number] and Fatigue_[specimen number], each representing an individual sample. Within each specimen directory, multiple subfolders are labeled with unique ID numbers, where each ID corresponds to a distinct tensile strain level or fatigue fracture progress state. Each subfolder contains the following files: (1) raw X-CT images in TIFF format, (2) binary-processed X-CT images highlighting internal defects in TIFF format, (3) 3D external and internal visualizations in JPG format, and (4) quantified defect metrics (birth and lifetime histogram) in JPG format.

The raw X-CT data typically comprises ~1,000 high-resolution image slices. The binary-processed images, refined to remove background noise, preserve only internal defect features for clarity. The 3D visualizations, rendered using Dragonfly software, enable a holistic view of both surface morphology and internal defect architecture. These images are particularly valuable for evaluating crack propagation pathways, void formation, and surface alterations post-deformation.

Additionally, histograms reflecting defect density and average inter-defect distances are included as advanced metrics. Defect density quantifies the number and volume of defects per unit volume, while distance metrics provide insight into spatial distribution. These quantitative tools, introduced in prior studies, offer new avenues for interpreting material integrity and damage evolution.

### Excel sheet

**Fig. 6:**
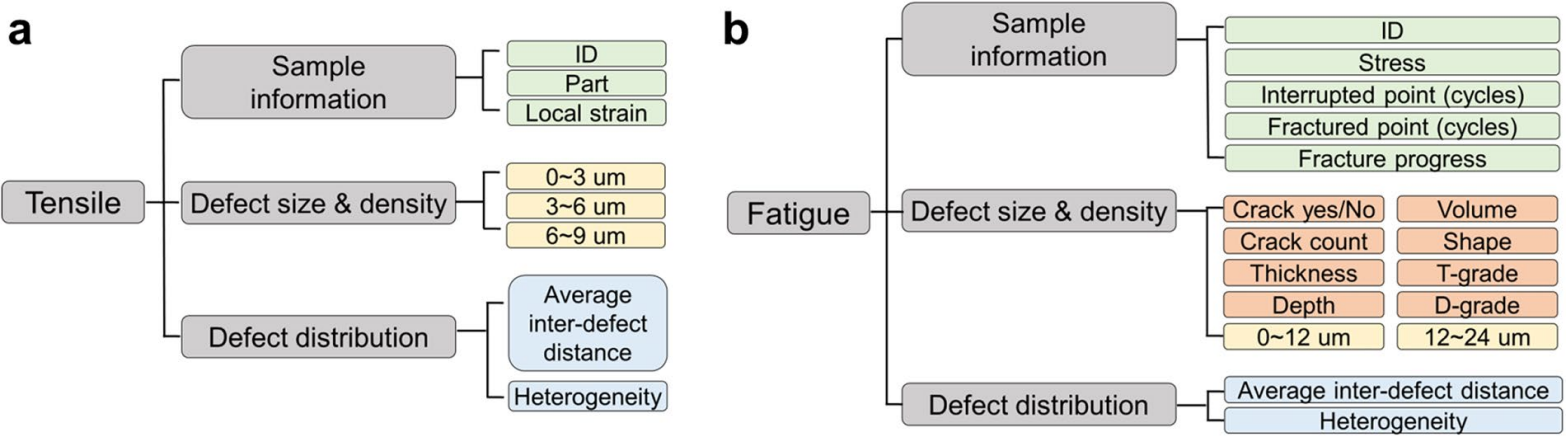
Structure of the Excel summary sheet. Each data type is color-coded: Light green for sample metadata, light yellow for defect size & density, light orange for crack-specific parameters, and light blue for defect distribution.

In addition to the structured data folders, the dataset^23^ includes a consolidated Excel sheet that tabulates all analyzed parameters from both tensile and fatigue experiments. The organization of this summary is depicted in Fig. 6.

For tensile data, the sheet includes sample metadata such as ID, part number (“Part”), and corresponding local strain. “Part” refers to the sub-region of the sample where strain was measured. Defect size and density are reported as the number density of defects (#/mm^3^) within three size categories: 0–3 μm, 3–6 μm, and 6–9 μm. Since cracks are not observed in tensile tests, only void-based parameters are provided. The defect distribution section summarizes average inter-void distances (D, μm) and heterogeneity (V, μm) to illustrate spatial defect dispersion, both derived from PH analysis. A higher D value indicates that voids are more widely spaced, whereas a higher V value reflects a more heterogeneous spatial distribution of voids.

**Fig. 7:**
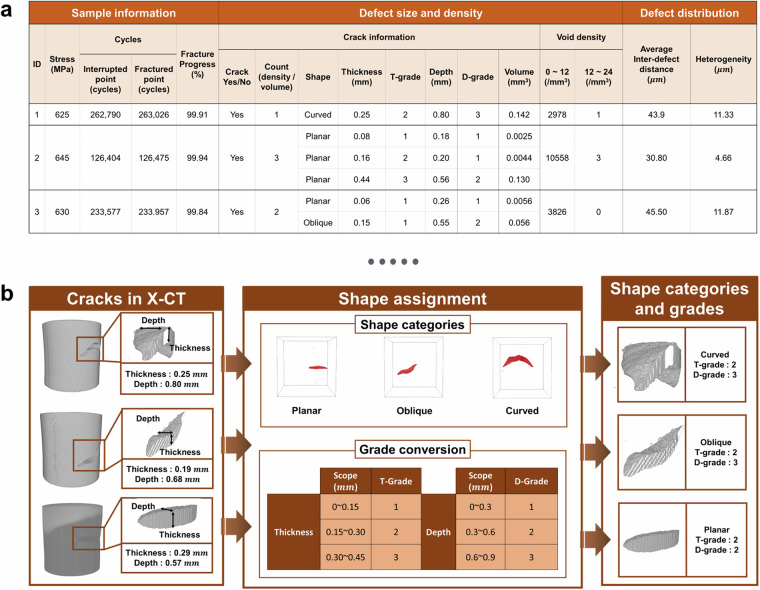
Example of an all-in-one table and grade converter. (**a**) Example excerpt from the Excel summary sheet showing fatigue sample data. (**b**) Schematic of crack classification logic by shape, thickness (T-grade), and depth (D-grade).

The fatigue data follows a similar format with additional complexity. Sample information includes applied stress (MPa), the number of cycles at interruption and at final fracture, and the corresponding fracture progress (%). Crack information is more detailed than in tensile datasets and includes presence/absence (Yes/No), crack count, reported as the number of cracks identified within the investigated volume, and qualitative shape classification. Quantitative crack descriptors comprise thickness (mm) within T-grade, depth (mm) with D-grade, and crack volume (mm^3^). Void characteristics are reported as density values (#/mm^3^) separated into two size categories (0–12 μm and 12–24 μm). In addition, defect distribution is characterized by the average inter-defect distance (D, μm) and heterogeneity (V, μm). An example of the fatigue Excel sheet is shown in Fig. 7a. In total, 16 descriptors are provided per sample.

Crack morphology classification is critical for understanding material degradation and predicting remaining service life. The dataset^23^ incorporates a two-stage scheme to categorize crack geometries, illustrated in Fig. 7b. Cracks are first grouped by propagation angle into three shapes: (1) planar (parallel to stress), (2) oblique (angled at 45°), and (3) curved (combinations of angled segments). Next, crack size is classified into thickness (T-grade) and depth (D-grade) bins, each split into three equal ranges derived from observed maxima. T-grades span 0–0.15 mm (Grade 1), 0.15–0.30 mm (Grade 2), and 0.30–0.45 mm (Grade 3). D-grades span 0–0.3 mm (Grade 1), 0.3–0.6 mm (Grade 2), and 0.6–0.9 mm (Grade 3). This classification system enables effective comparison and quantification of crack features across specimens.

### Data Overview

Table 1 summarizes the distribution of the 276 datasets, comprising 134 tensile and 142 fatigue datasets. The tensile datasets are distributed across seven local strain ranges from 0–20% to >120%. The fatigue datasets are categorized into high-cycle (66 datasets), low-cycle (51 datasets), and ultralow-cycle (35 datasets), and further subdivided by fracture progress intervals (0–20%, 20–40%, 40–60%, 60–80%, 80–90%, 90–95% and 95–100%). This stratification provides a clear overview of how the dataset covers different deformation and fracture regimes.

**Table 1:** Data distributions.

Tensile							
**Local strain (%)**	**0–20**	**20–40**	**40–60**	**60–80**	**80–100**	**100–120**	**120-**
**Number of datasets**	37	32	13	6	13	23	10
**Fatigue**							
**Fracture progress (%)**	**0–20**	**20–40**	**40–60**	**60–80**	**80–90**	**90–95**	**95–100**
**Number of datasets (high-cycle)**	9	11	0	3	7	6	28
**Number of datasets (low-cycle)**	2	10	11	9	9	4	3
**Number of datasets (ultralow-cycle)**	0	5	4	16	5	0	0

This summarizes the distribution of the 276 collected datasets^23^, comprising 134 tensile and 142 fatigue datasets^23^. The fatigue data is further categorized into 64 high-cycle, 48 low-cycle, and 30 ultralow-cycle fatigue datasets^23^.

As shown in Figs. 6, 7, each defect feature contains multiple image types: 2D X-CT image sets, 2D X-CT image sets processed with window leveling, 3D reconstructed image, birth histogram and lifetime histogram. For the tensile defect feature, a total of 670 image data files were collected. Additionally, each of the 134 tensile datasets^23^ includes 7 quantified parameters (sample ID, local strain, defect density in three size categories, D-value and V-value), resulting in 938 total data points.

For the fatigue defect feature, 710 image files were generated. Each of the 117 crack-free features contains 10 quantified parameters, while the 25 crack-containing datasets^23^ include 17 parameters. This yields 1,170 and 425 data points, respectively, totaling 2,305 data points from the fatigue experiments.

## Technical Validation

**Fig. 8:**
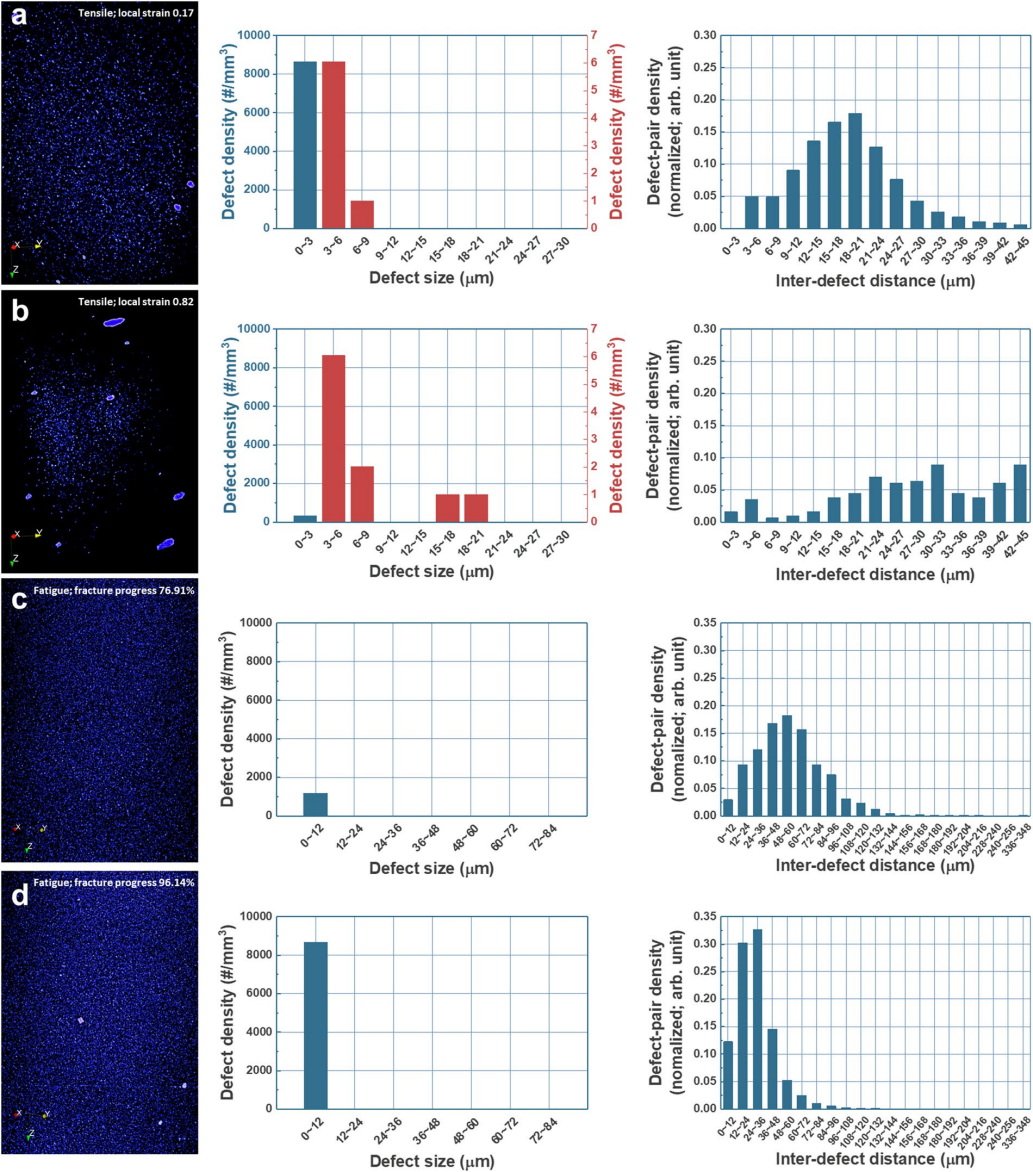
Representative internal defect images and metrics. (**a,b**) Tensile samples at local strain 0.17 and 0.82. (**c,d**) Fatigue samples at 76.91% and 96.14% fracture progress. Red dots indicate internal defects. Associated plots show defect density by size and pair-density by distance.

As mechanical damage progresses, the size, density, and spatial distribution of internal defects evolve significantly. Figure 8 presents representative examples from both tensile and fatigue datasets^23^. Figure 8a,b correspond to tensile data from the same specimen but captured at different local strains. At low local strain (Fig. 8a), numerous small voids are visible, and the distribution of defect-pair distances follows a normal distribution. At higher local strain (Fig. 8b), larger defects become more prominent, and the distribution of inter-defect distances becomes more irregular. This reflects the typical sequence of damage in tensile deformation: homogeneous formation of small voids, growth and coalescence of these voids, and eventual nucleation of larger cracks as inhomogeneities form.

Figure 8c,d show fatigue data acquired at different stages of fracture progression from the same specimen. In early-stage fatigue (Fig. 8c), only small internal defects are visible, and their spatial distribution follows a normal curve. In contrast, late-stage fatigue (Fig. 8d) shows an abundance of large internal defects in the 3D view, although the size-density plot misleadingly classifies them in smaller categories due to a limitation in the software’s pixel handling capacity. Specifically, while the raw X-CT scans were acquired at a resolution of 3 µm, the statistical analyses were performed on downscaled data with an effective voxel size of 12 µm to overcome software memory constraints. This downscaling resulted in broader defect classification bins without altering the raw scan resolution. Based on statistical validation of the downscaled dataset, defects with their longest axis ≥36 µm (≥3 voxels at 12 µm) were consistently identified as cracks, and this threshold was adopted as the minimum crack size criterion in the fatigue analysis. Despite these limitations, the overall defect evolution remains clearly discernible, as a significant increase in defect quantity is observed and the inter-defect pair distance curve becomes narrower and shifts forward with fracture progression.

**Fig. 9:**
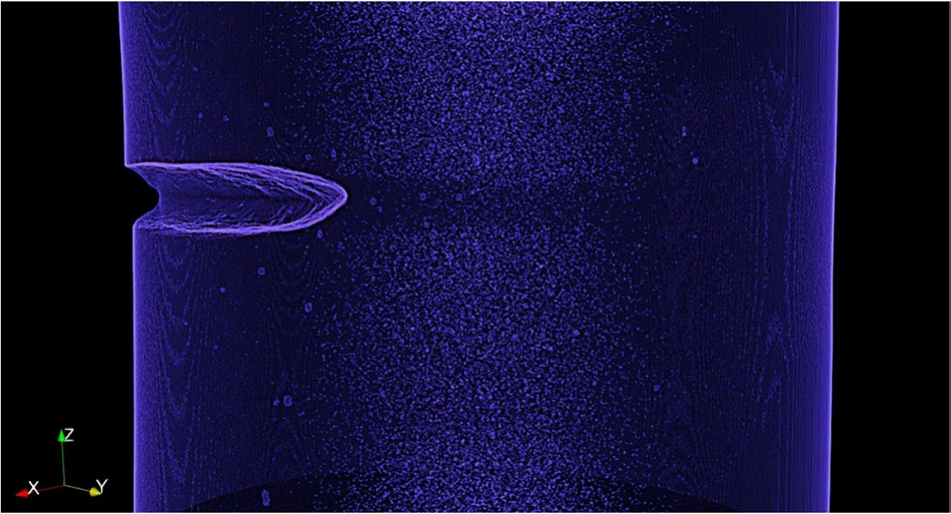
3D X-CT image of a fatigue sample with 99.8% fracture progress, illustrating a surface crack surrounded by large defect clusters.

Figure 9 provides a 3D visualization of a fatigue specimen at 99.8% fracture progress. The surface crack is clearly visible, and a dense distribution of defects, particularly larger ones, can be observed around the crack zone. This highlights how surface crack propagation correlates with surrounding defect coalescence. Under cyclic loading, stress concentrations initiate intrusions and extrusions on the surface, which evolve into cracks^28,29^. Simultaneously, repeated loading promotes the internal development of microdefects^7,30^. Once cracks initiate, stress localizes at their tips, accelerating both their growth and the coalescence of nearby voids^31,32^. As crack growth progresses, the specimen’s effective cross-sectional area decreases, intensifying true stress and accelerating deformation^33^, an effect evident in the final rapid rise in displacement seen in Fig. 3.

**Fig. 10:**
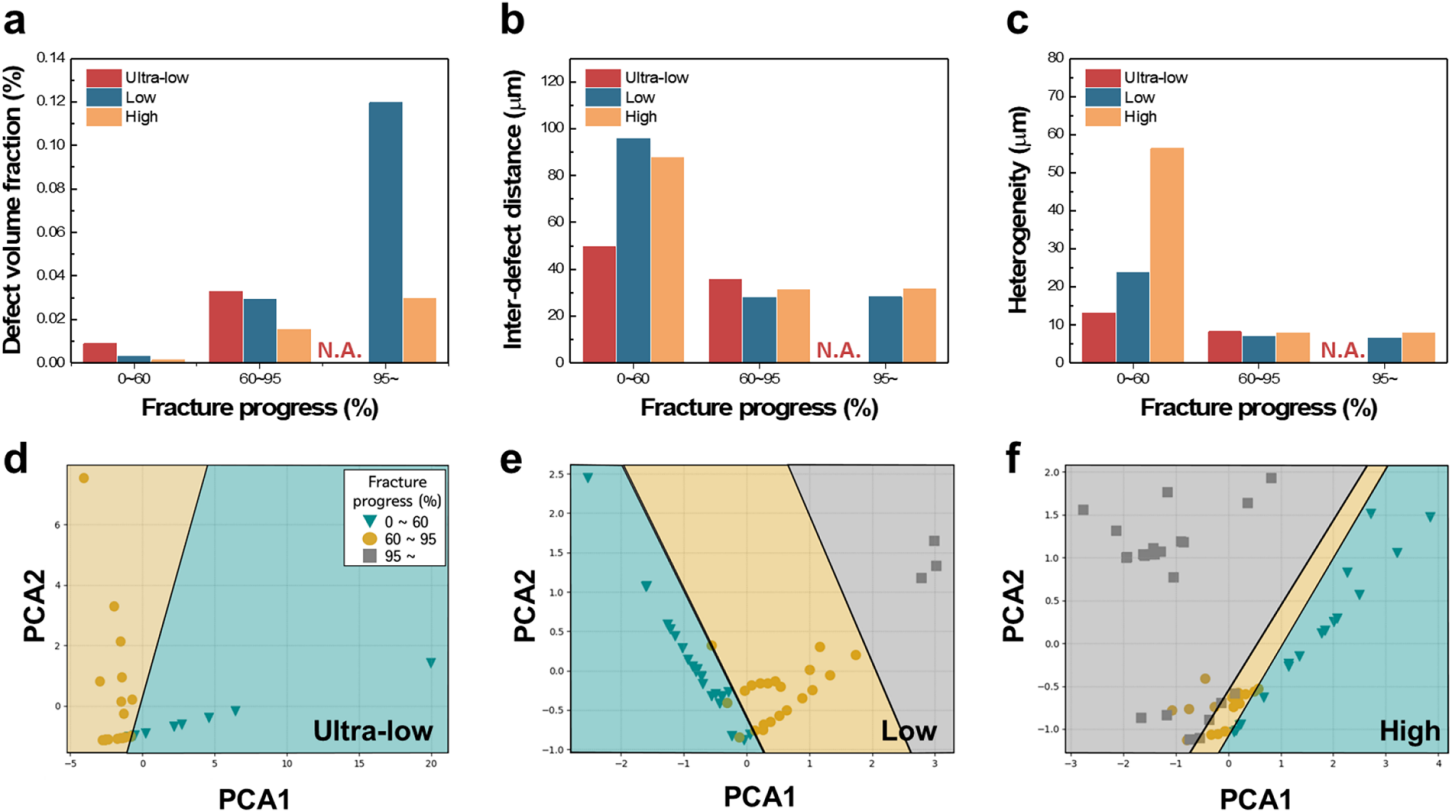
Evolution of internal defect metrics across fracture progression. (**a**) Total defect volume. (**b**) Inter-defect distance. (**c**) Heterogeneity. (**d**–**f**) PCA clustering of defect parameters, revealing distinct groupings by fatigue mode.

Figure 10 summarizes the quantitative defect metrics across three fracture progress levels (low: 0–60%, middle: 60–80%, high: 80–100%) for high-, low-, and ultralow-cycle fatigue groups. As fracture progresses, the total defect volume increases (Fig. 10a), while both average inter-defect distance and heterogeneity decrease (Fig. 10b,c). This indicates the continuous formation of new microdefects and coalescence of existing ones. Notably, ultralow-cycle fatigue shows consistently higher defect volumes across the entire progression spectrum^34^. In contrast, high-cycle fatigue only reaches significant defect accumulation near failure^34^.

Finally, cluster analysis was performed to validate the consistency and significance of the extracted defect features. Four-dimensional vectors, representing number of cracks and voids, heterogeneity, and average inter-defect distance, were used as input. Principal Component Analysis (PCA) was applied for dimensionality reduction, followed by clustering. This analysis successfully separated samples into three groups consistent with high, low, and ultralow-cycle fatigue (Fig. 10d–f), affirming the effectiveness of the quantitative descriptors in representing damage evolution.

Although the present dataset^23^ was generated from a low-alloy ferritic steel with a specific thermomechanical treatment, the experimental framework and data analysis pipeline are broadly applicable to steels with different compositions and processing histories. The fundamental processes of void nucleation, growth and coalescence under tensile and fatigue loading are common across a wide range of steels, even though the precise kinetics and defect morphologies may vary with alloy chemistry and microstructure. Therefore, while direct transfer of the defect statistics to other steel grades should be made with caution, the dataset^23^ provides a valuable methodological and reference baseline that can be extended or adapted to other material systems.

## Data Availability

The dataset is available at Dryad (10.5061/dryad.9cnp5hqpf). It includes raw and binary-processed X-CT images, metadata files, and quantified descriptor of internal defects from both tensile and fatigue experiments.

## Code availability

To preprocess and analyze the X-CT images, we employed Dragonfly (Object Research Systems, Inc.), a commercial software platform specialized for image processing and segmentation. For the computation of persistent homology and topological feature extraction, we utilized the HomCloud library (https://homcloud.dev). The Python source codes used to generate the dataset, including image processing, persistent homology computation are publicly available at the following GitHub repository: https://github.com/KIST-CSRC/material-failure-prediction.
